# Hypogonadotropic Hypogonadism and Short Stature in Patients with Diabetes Due to Neurogenin 3 Deficiency

**DOI:** 10.1210/jc.2016-2319

**Published:** 2016-08-17

**Authors:** Oscar Rubio-Cabezas, José Luis Gómez, Andrea Gleisner, Andrew T. Hattersley, Ethel Codner

**Affiliations:** Department of Pediatric Endocrinology (O.R.-C.), Hospital Infantil Universitario Niño Jesús, Instituto de Investigación Sanitaria La Princesa, 28007 Madrid, Spain; Department of Pediatrics (J.L.G.), Complejo Hospitalario Torrecárdenas, 04009 Almería, Spain; Department of Pediatrics (A.G.), University of Concepcion, School of Medicine, 4070038 Concepción, Chile; Institute for Biomedical and Clinical Science (A.T.H.), University of Exeter Medical School, Exeter EX2 5DW, United Kingdom; and Institute of Maternal and Child Research (E.C.), School of Medicine, University of Chile, 8360160 Santiago, Chile

## Abstract

**Context:**

Biallelic mutations in *NEUROG3* are known to cause early-onset malabsorptive diarrhea due to congenital anendocrinosis and diabetes mellitus at a variable age. No other endocrine disorders have been described so far. We report four patients with homozygous *NEUROG3* mutations who presented with short stature and failed to show any signs of pubertal development.

**Case Description:**

Four patients (two males, two females) were diagnosed with homozygous mutations in *NEUROG3* on the basis of congenital malabsorptive diarrhea and diabetes. All four had severe short stature and failed to develop secondary sexual characteristics at an appropriate age, despite some having normal body mass index. The absence of gonadal function persisted into the third decade in one patient. Upon testing, both basal and stimulated LH and FSH levels were low, with the remaining pituitary hormones within the normal range. Magnetic resonance imaging scans of the hypothalamic-pituitary axis did not reveal structural abnormalities. A diagnosis of hypogonadotropic hypogonadism was made, and replacement therapy with sex hormones was started.

**Conclusions:**

The high reproducibility of this novel phenotype suggests that central hypogonadism and short stature are common findings in patients with mutations in *NEUROG3*. Growth rate needs to be carefully monitored in these patients, who also should be routinely screened for hypogonadism when they reach the appropriate age. *NEUROG3* mutations expand on the growing number of genetic causes of acquired hypogonadotropic hypogonadism.

## Abstract (precis)

Patients with neurogenin 3 deficiency show congenital diarrhea and diabetes. We describe four patients who showed hypogonadotropic hypogonadism, short stature and diabetes due to mutations in NEUROG3

Biallelic mutations in *NEUROG3* result in severe congenital malabsorptive diarrhea due to a lack of enteroendocrine cells in the gut (1) and diabetes with a variable age of onset, in keeping with the role of neurogenin 3 in pancreatic endocrine differentiation during embryonic development (1–3). No other endocrine manifestations have been described to date. We describe four patients with two different homozygous mutations in *NEUROG3* who have hypogonadotropic hypogonadism and short stature, and we elaborate on the potential mechanisms underlying these associations.

## Case Reports

The study was conducted in accordance with the Declaration of Helsinki, and all of the patients gave written informed consent. The genetic findings and details of the diabetes have been previously described (3).

Details of development and endocrine investigations (Table 1) and growth charts (Supplemental Data) of all four patients are provided.

**Table 1.** Clinical Findings and Hormonal Profiles of the Patients

	Patient 1	Patient 2	Patient 3	Patient 4
Gender	Female	Male	Female	Male
Age, y	19.6	18	15.5	19
Bone age, y	13	12	13	14
Height, cm	144	133.0	132.0	143.0
Height (SDS)	−3.0	−5.7	−4.7	−4.6
Mean parental height (SDS)	−1.7	−1.0	−1.4	−1.4
Weight, kg	46	30.6	32.0	36.6
BMI, kg/m^2^	22.2	17.3	18.4	17.9
BMI (SDS)	0.2	−2.5	−0.7	−2.3
*NEUROG3* gene mutation	L135P/L135P	L135P/L135P	R107S/R107S	R107S/R107S
Age at diabetes diagnosis, y	0.05^a^	13	12	24
Basal LH/FSH, mIU/mL	0.15/1.7	0.03/0.48	0.10/0.2	0.10/1.4
Gonadotropin stimulation test	GnRH analog (100 μg)	GnRH analog (100 μg)	Leuprolide acetate (500 μg)	Leuprolide acetate (500 μg)
Peak stimulated LH/FSH, mIU/mL	0.52/6.8	0.93/3.52	1.2/n.d.	1.6/n.d.
Estradiol, pg/mL	Below detection limit of the assay	n.d.	10.4	n.d.
T, ng/mL	0.26	0.17	n.d.	0.22
IGF-1, ng/mL	265	139	236	107
IGF-1 SDS	0.19	−1.58	−0.22	−2.0
IGFBP-3, mg/L	2.7	3.5	3.5	2.6
IGFBP-3 SDS	−1.53	0.0	−0.79	−1.2
Pubertal stage at the time of IGF-1 and IGFBP-3 sampling	Prepubertal	Prepubertal	Prepubertal	Prepubertal
Hypothalamic-pituitary imaging	Normal MRI (1.5 T, 3 mm slice)	Normal CT scan	Normal MRI (1.5 T, 2 mm slice)	Normal MRI (1.5 T, 2 mm slice)
Other clinical features	Genu valgum, cubitus valgum, short neck	Genu valgum		Mild left hemiparesis

Abbreviations: CT, computed tomography; n.d., not determined.

a This patient had transient neonatal diabetes that relapsed at 6 years of age.

### Patient 1

A female patient was diagnosed with a homozygous L135P mutation in *NEUROG3* identified on the basis of congenital diarrhea and remitting and relapsing transient neonatal diabetes. On investigation at 20 years of age, she had a complete absence of pubertal development and short stature (−3.3 SD score [SDS]). Her body mass index (BMI) was 19.8 kg/m^2^. Isolated hypogonadotropic hypogonadism was diagnosed based on the lack of breast development and prepubertal internal genitalia on pelvic ultrasound and on basal and stimulated gonadotropin and estradiol levels in the prepubertal range. Hypothalamic-pituitary magnetic resonance imaging (MRI) was normal. Ethinyl estradiol 5 mg/d was started and was increased stepwise. Progesterone was added later. Serum levels of IGF-1, IGF binding protein-3 (IGFBP-3), thyroid hormones, dehydroepiandrosterone sulfate, and prolactin were normal. GH treatment was used for 1 year, but no increase in growth velocity was observed. Currently, the patient is 23 years old, and her present height is 146 cm (−2.7 SDS).

### Patient 2

A male patient was diagnosed with a homozygous L135P mutation in *NEUROG3* identified on the basis of congenital diarrhea and diabetes at 13 years of age. At 18 years, he had a prepubertal penis, a testicular volume of 2 mL bilaterally, markedly short stature (−5.7 SDS), and a low BMI of 16.6 kg/m^2^. His bone age was 12 years. Laboratory results showed low gonadotropins that did not respond to exogenous GnRH administration. A hypothalamic-pituitary computed tomography scan was normal. A stimulation test for GH with clonidine showed normal stimulated GH levels (peak, 9.8 ng/mL). GH administered for 1 year did not improve growth velocity. The patient is now 19 years old, and puberty is being treated with an increasing dose of T enanthate.

### Patient 3

A female patient born to consanguineous parents was diagnosed with a homozygous R107S mutation in *NEUROG3* identified on the basis of congenital diarrhea and diabetes at 14 years of age. At 15.5 years, she was evaluated for primary amenorrhea. Physical examination revealed breast and pubic hair Tanner stage 2, short stature (−4.7 SDS), and normal BMI (18.4 kg/m^2^). Her bone age was 13 years. Laboratory tests demonstrated low gonadotropins that did not rise after stimulus with a long-acting GnRH analog. IGF-1, IGFBP-3, basal GH, adrenal steroids, thyroid, and prolactin levels were normal. Hypothalamic-pituitary MRI was normal. Puberty was induced using transdermal estradiol given in patches in progressive doses for 2 years, before progestagens were added to the treatment. After induction, pubertal stage progressed to S4P4, and she started having regular menses at 17.9 years of age. Her current height is 141.2 cm (−3.8 SDS) at 20 years of age.

### Patient 4

Patient 4 was the older male sibling of patient 3. He had the same homozygous R107S mutation in *NEUROG3* and congenital diarrhea, but he did not develop diabetes until 24 years of age. At 19 years, he was evaluated for the lack of pubertal development. Physical examination showed his penis was 6 cm in length, testicular volume was 3 mL bilaterally, and pubic hair was scarce. He had short stature (−4.6 SDS) and a BMI of 17.9 kg/m^2^. His bone age was 14 years. Laboratory tests demonstrated low gonadotropins that did not rise after stimulus with a long-acting GnRH analog. IGF-1, IGFBP-3, thyroid and adrenal steroid levels were normal. Hypothalamic-pituitary MRI was normal. Puberty was induced with treatment with LH in increasing doses (1–2000 IU) over 18 months until a testicular volume of 8 mL was reached when FSH 75 IU was added. At the end of induction, testicular volume had increased to 12 mL, penis length increased to 9 cm, and T cypionate was given in increasing doses. The patient's current height is 151.3 cm (−4.0 SDS).

## Conclusions

Biallelic mutations in *NEUROG3* are known to cause a rare but well-defined clinical syndrome characterized by severe malabsorptive diarrhea from early life and mild nonketotic diabetes with a variable age of onset (1, 2). The novel and consistent association of persistent hypogonadotropic hypogonadism in these patients suggests that the expression of *NEUROG3* in the hypothalamus also plays an unexpected role in postnatal pubertal development.

Among the variety of mechanisms potentially explaining the central hypogonadism in these patients, secondary leptin deficiency due to chronic malnutrition is unlikely, given the normal BMI in two of the patients. An alternative explanation might be related to the lack of systemic incretin hormones, mainly GLP-1, because a delayed onset of puberty has been previously reported in mice with enteroendocrine hormone deficiency (4). However, the more likely explanation relates to local expression of *NEUROG3* in the hypothalamus.

It remains currently undetermined whether neurogenin 3 plays a role during normal development or functioning of GnRH-producing neurons in humans. Nevertheless, its expression in the ventromedial and arcuate (ARC) nuclei of the anterior hypothalamus has been repeatedly reported in animal models, where it is required for the correct specification of neuronal subtypes controlling energy homeostasis. Up-regulation of the hypothalamic orexigenic neuropeptide Y (NPY) system is a feature characteristic of *Ngn3* null mice (5). Because NPY plays a role in the regulation of Kiss1 (6), a lack of *Ngn3* leading to chronic elevations of NPY may explain a dysregulation in Kiss1, explaining the hypogonadotropic hypogonadism observed in our series. In addition to the increased activity of NPY neurons, *Ngn3* mutants display a loss of *Pomc* expression in the ARC that is associated with postnatal obesity and a loss of leptin sensitivity (7, 8). In keeping with this, *Pomc*-expressing progenitors also differentiate into ARC-localized Kiss1 neurons (9). Furthermore, direct apposition between GnRH-containing axon terminals and POMC cell bodies suggests a possible functional relationship between these two neuronal types (10).

Severe short stature (−3 to −5.7 SDS) was observed in all four subjects, which has not been reported previously in animal models of *NEUROG3* deficiency. The growth charts suggest that the reduced stature is seen by 6–7 years of age in all cases (Supplemental Data). The etiology of the reduced stature is uncertain. Growth hormone deficiency is unlikely because IGF1 and IGFBP-3 were within normal range, one patient had a normal stimulated GH and the two cases were treated with growth hormone did not have a significant increase in growth velocity. The mid-parental height of −1 to −1.7 SDS is consistent with a modest influence of a heterozygous mutation.

In summary, our data suggest that *NEUROG3* deficiency may impair pubertal development and growth, in addition to its well-established effects on gastrointestinal and pancreatic endocrine function. Further studies are needed to clarify the exact mechanism responsible for this clinical observation.
